# Mesomelia-synostoses syndrome: contiguous deletion syndrome, *SULF1* haploinsufficiency or enhancer adoption?

**DOI:** 10.1186/s13039-024-00684-2

**Published:** 2024-07-12

**Authors:** Ingrid Bendas Feres Lima, Lúcia de Fátima Marques de Moraes, Carlos Roberto da Fonseca, Juan Clinton Llerena Junior, Mana Mehrjouy, Niels Tommerup, Elenice Ferreira Bastos

**Affiliations:** 1 Clinical Cytogenetics Laboratory, Center for Medical Genetics/IFF/Fiocruz, Rio de Janeiro, Brazil; 2 Reference Center for Rare Diseases/IFF/Fiocruz, Rio de Janeiro, Brazil; 3 Department of Cellular and Molecular Medicine, University of Copenhagen, Copenhagen, Denmark

**Keywords:** Balanced translocations, *GABRA5*, Mesomelia-Synostoses syndrome, *SLCO5A1*, *SULF1*; enhancer adoption

## Abstract

**Background:**

Mesomelia-Synostoses Syndrome (MSS)(OMIM 600,383) is a rare autosomal dominant disorder characterized by mesomelic limb shortening, acral synostoses and multiple congenital malformations which is described as a contiguous deletion syndrome involving the two genes *SULF1* and *SLCO5A1*. The study of apparently balanced chromosomal rearrangements (BCRs) is a cytogenetic strategy used to identify candidate genes associated with Mendelian diseases or abnormal phenotypes. With the improved development of genomic technologies, new methods refine this search, allowing better delineation of breakpoints as well as more accurate genotype-phenotype correlation.

**Case presentation:**

We present a boy with a global development deficit, delayed speech development and an ASD (Asperger) family history, with an apparently balanced “de novo” reciprocal translocation [t(1;8)(p32.2;q13)dn]. The cytogenetic molecular study identified a likely pathogenic deletion of 21 kb in the 15q12 region, while mate pair sequencing identified gene-truncations at both the 1p32.2 and 8q13 translocation breakpoints.

**Conclusions:**

The identification of a pathogenic alteration on 15q12 involving *GABRA5* was likely the main cause of the ASD-phenotype. Importantly, the chr8 translocation breakpoint truncating *SLCO5A1* exclude *SLCO5A1* as a candidate for MSS, leaving *SULF1* as the primary candidate. However, the deletions observed in MSS remove a topological associated domain (TAD) boundary separating *SULF1* and *SLCO5A1.* Hence, Mesomelia-Synostoses syndrome is either caused by haploinsufficiency of *SULF1* or ectopic enhancer effects where skeletal/chrondrogenic *SULF1* enhancers drive excopic expression of developmental genes in adjacent TADs including *PRDM14, NCOA2* and/or *EYA1*.

## Background

The characterization of chromosomal rearrangements, especially balanced translocations associated with an abnormal phenotype, is a classical strategy for delineation of candidate regions/genes associated with Mendelian diseases [1–3]. Examples are Duchenne Muscular Dystrophy, Neurofibromatosis type 1, BPES syndrome, and many others [4–6]. With technological advances in molecular methods, such as Microarray Comparative Genomic Hybridization (aCGH) and Next-Generation Sequencing (NGS) paired-end sequencing techniques, genotype-phenotype relationship can be even more precisely revealed [3, 7].

Mesomelia-Synostoses syndrome (MSS) or Verlores-David-Pfeiffer syndrome (VDPS) is a rare autosomal dominant disease characterized by mesomelic limb shortening combined with multiple congenital malformations and typical acral synostoses [8, 9]. With one apparent exception, affected individuals have a deletion in the 8q13 region comprising two closely located genes, *SULF1* and *SLCO5A1* [10, 11]. Hence, MSS is characterized as a contiguous deletion syndrome in OMIM (#600,383).

Autism Spectrum Disorder (ASD) is a complex neurological disorder with strong genetic influence. Hundreds of genes associated with a high risk for ASD have been identified [12], but the etiology of the disease is still unknown in most cases.

We present a boy with ASD and dysmorphisms, with an apparently balanced “de novo” translocation t(1;8)(p32.2;q31.3)dn. Mate-pair sequencing identified a disruption of *SLCO5A1*. The patient does not have any MSS features, supporting that loss-of-function of *SLCO5A1* is not a determining factor in MSS.

### Case presentation

The 4 years old boy was referred for genetic evaluation due to developmental delay, language delay and a family history of autism (Asperger syndrome). The parents were non-consanguineous. An older brother and a maternal cousin have Asperger’s, the latter with language deficit with normal auditory processing. There was an adequate and normal motor development. There was a deficit in verbal and non-verbal communication, with syllable speaking at age 3 and an inability to form sentences. He does not understand verbal orders and lacks autonomy. Otherwise he had normal anthropometric and growth data within the family’s growth curve.

**Fig. 1:**
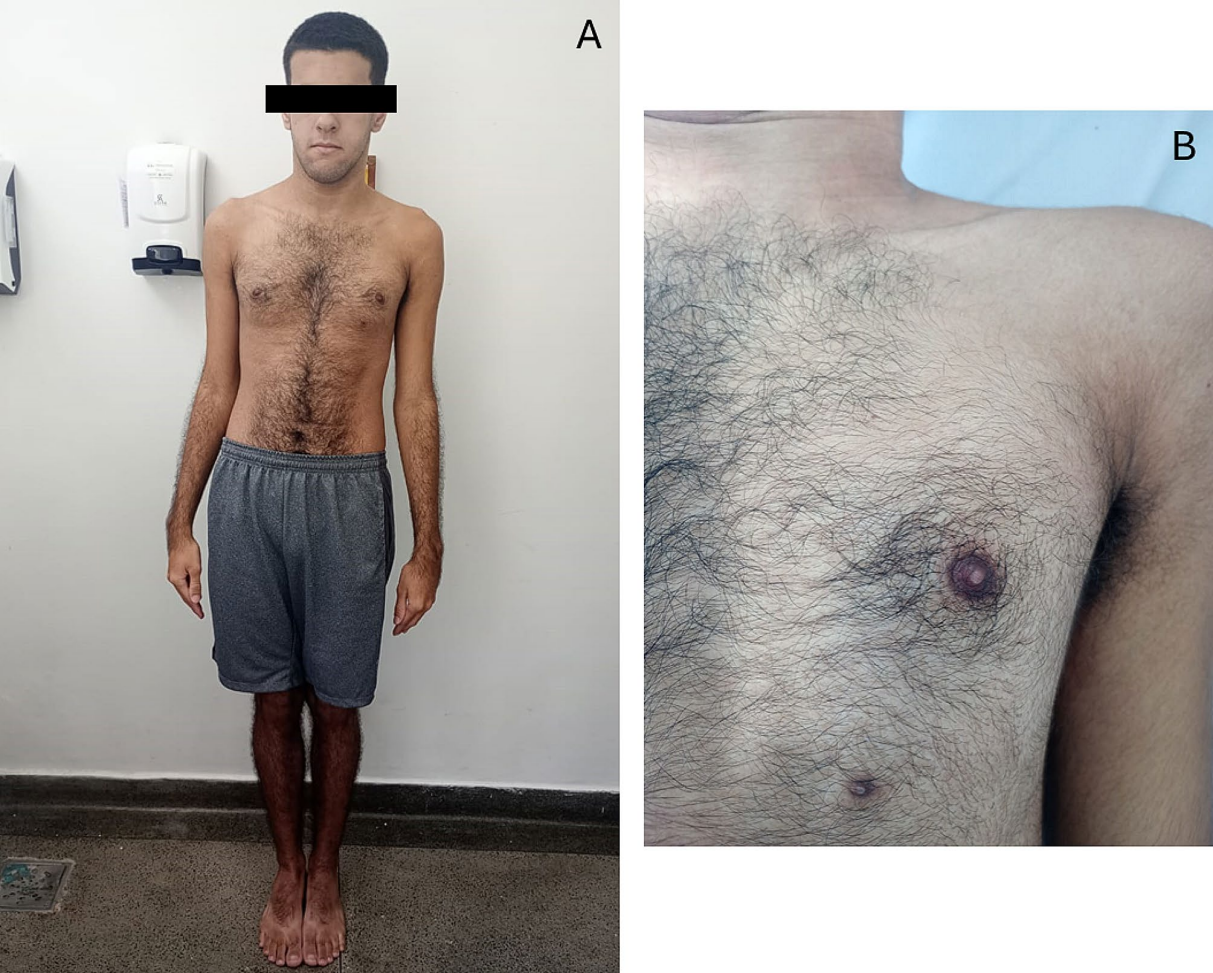
**(A)** Longilineal patient, torso and limbs longer than average **(B)** Polytheia, supernumerary nipple on the left side

The patient was re-evaluated at the age of 16, presenting with social phobia, no socialization and hyperacusis. On physical examination, he presented left side polythelia, long-limbed and no dysmorphic features. (Fig. 1)

### Methods

This study was approved by the Ethics Committee of Fernandes Figueira Institute (Fiocruz – CAEE 35766720.7.0000.5269), with written informed consent from the patient’s legal guardians.

### Cell culture, G-band karyotype analysis

The proband’s cytogenetic study was performed from peripheral blood stimulated with phytohemaglutinin. The cytogenetic analysis was carried out after GTG banding (450 band level) and the result was classified according to ISCN 2020 [13].

### Fluorescence in situ hybridization analysis (FISH)

The FISH technique was performed according to standard protocols, using cMYC (MYC) Breakapart (8q24.21 region), 1p36 (1p36.32 and 1qter region) and WCP8-specific gene (whole chromosome) probes (Cytocell, Inc.).

### Array CGH

Array CGH was performed using CytoScan®750 Affymetrix according to the manufacturer’s protocol. The analysis was performed using the Chromosome Analysis Suite program (Affymetrix®). The reference sequence used was Human Genome Build hg19 (UCSC Genome Browser February 2009).

### Mate-pair sequencing

Mate-pair sequencing was performed by the Department of Cellular and Molecular Medicine, University of Copenhagen using Illumina’s Nextera DNA library and Mate-pair sample preparation kit following the instructions. The final libraries were quantified using Pico Green (Quant-iT, Invitrogen) and Agilent Bioanalyzer DNA 1000 kit (Quant-iT, Invitrogen). Samples were sequenced on a HiSeq®2500 (Illumina, San Diego, CA, USA) (2 × 100 bp) and alternatively sequenced on an Illumina NextSeq 500 platform (2 × 75 or 2 × 150 bp). The mate-pairs passing Illumina Chastity filtering (> 0.6) were aligned to the hg19 human reference genome using Burrows-Wheeler-Aligner (BWA). Reads with unexpected strand orientation or reads aligning to different chromosomes were extracted and SVDetect and Delly software were used to detect potential rearrangements.

### Constraint scores in gnomAD

Loss-of function scores (pLI-scores) of *SULF1*, *SLCO5A1, PRDM14, NCOA2* and *EYA1* were obtained from gnomAD (https://gnomad.broadinstitute.org/).

### Topological Associated Domains (TADs) and chromatin loops

We obtained published coordinates for Topological Associated Domains (TADs) [14] and chromatin loops [15, 16].

## Results

### Cytogenetic analysis

**Fig. 2:**
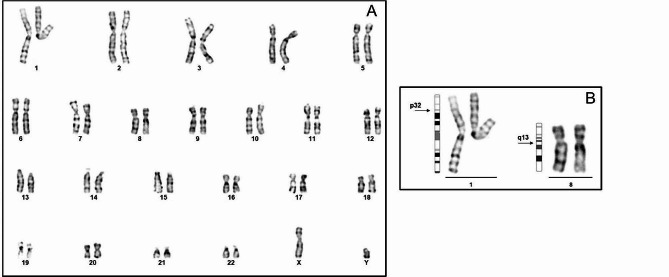
**(A)** Karyogram with 46,XY, t(1;8)(p32.2;q13)dn **(B)** Partial karyogram showing the balanced translocation with arrows at the breakpoints

Cytogenetic analysis identified a male karyotype with an apparently balanced translocation involving chromosome 1 and chromosome 8 [46, XY t(1;8)(p32.2;q13)]dn (Fig. 2).

### Fluorescence in situ hybridization-FISH

**Fig. 3:**
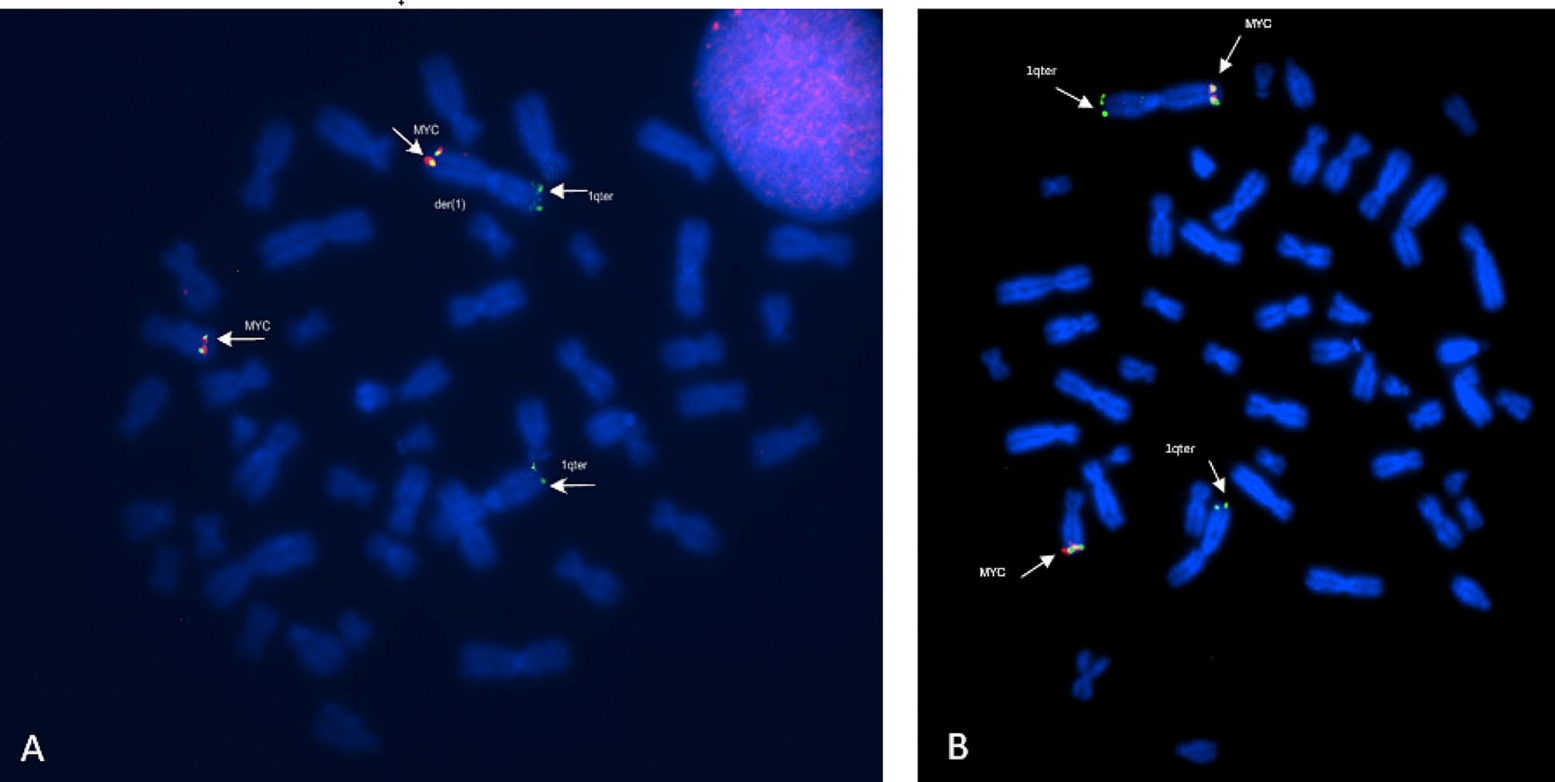
**A, B)** Result of FISH analysis using the probes MYC (red and green staining) and 1p36 pobre (1qter green staining), demonstrating the presence of the region 8q24.21 (MYC) on the der [1] chromosome

**Fig. 4:**
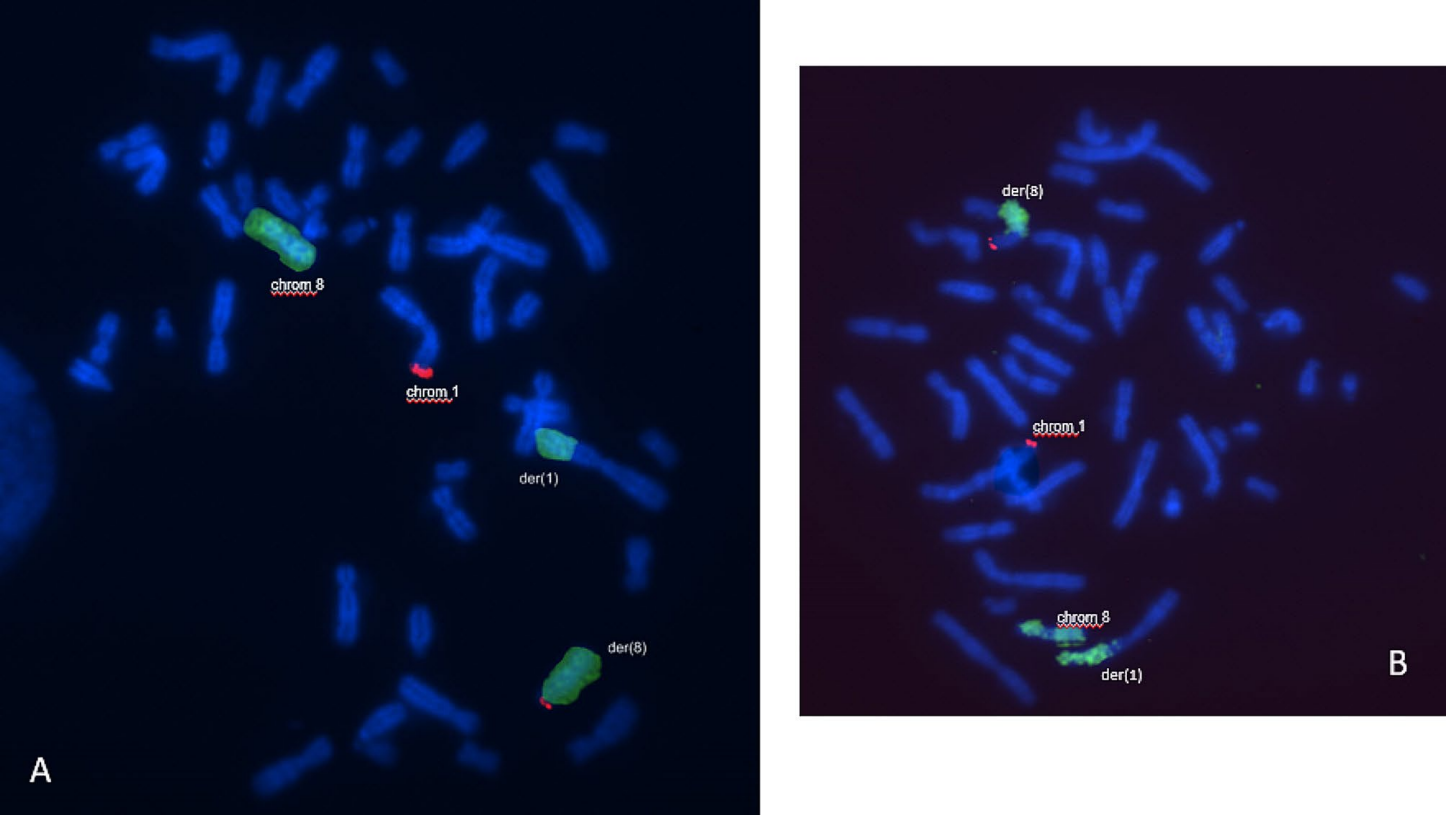
**A, B)** Result of FISH analysis using WCP8 probes - painting of 8 (green) and 1p36 (1p36.33 red). We can observe three green signals, one on the intact chromosome 8, and signals on both the der [8] and der [1] chromosomes. There are two red signals, one on the telomeric region of the innermost normal chromosome 1 and another on the der [8], which demonstrates the translocation between chromosomes 1 and 8

FISH delimited the breakpoints at t(1;8)(p36.3;q21) (Figs. 3 and 4).

### aCGH

Microarray analysis revealed a 21 kb deletion in 15q12 (46,XY.arr[GRCh19] 15q12 (27151153_27173084)x1) partially involving the coding region of the *GABRA5* gene. This is considered to be a pathogenic variant. No losses or gains were identified in the regions involved in the chromosomal rearrangement.

### Mate-pair sequencing

**Fig. 5:**
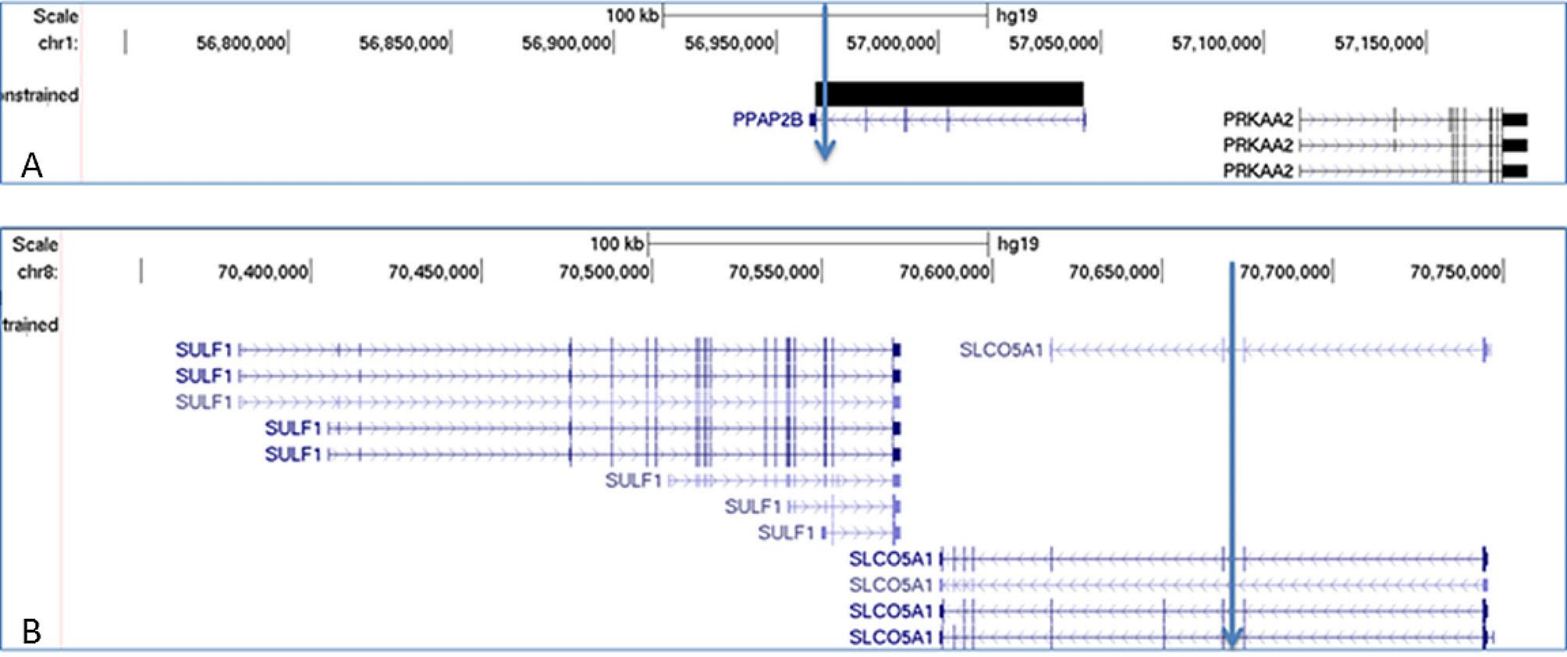
Diagram demonstrating the breakpoint regions based on the results from the mate-pair analysis 1 **(A)** chromosome 1 with the breakpoint within *PPAP2B* and **(B)** chromosome 8 where the break occurred in the *SLCO5A1* gene

Mate-pair sequencing identified a breakpoint on chromosome 1 (chr1:56964825_56965128 (hg19)) within the first exon of *PLPP3* (phospholipid phosphatase 3 (Fig. 5A). The breakpoint on chromosome 8 (chr8:70669917_70670110) disrupted *SLCO5A1* (Fig. 5B). There were no additional breakpoints in the genome, nor reduced coverage of paired-end reads in the breakpoint regions, compatible with the normal array-CGH-results. Hence, the t(1;8) is a simple two-way balanced reciprocal translocation.

**Fig. 6:**
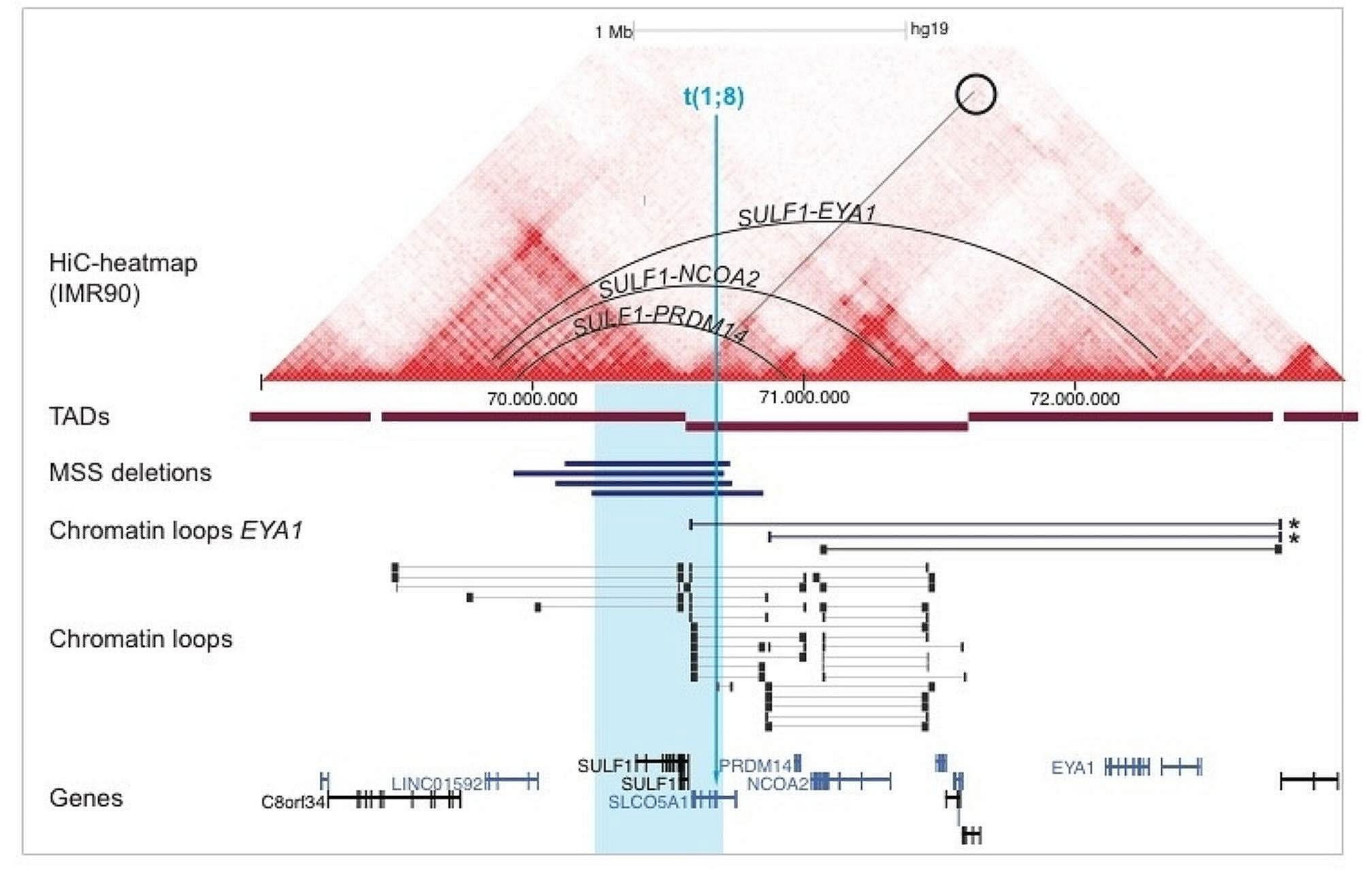
Genome Browser view of a 4 Mb region in 8q13, showing the reported deletions in MSS (bars and blue segment) and the t(1;8) breakpoint truncating *SLCO5A1* (arrow), in the context of HiC chromatin loops [15] and HiC-heatmaps defining TADs [14]. *Denotes loops defined by micro-C [16]. The MSS deletions remove a TAD boundary between *SULF1* and *SLCO5A1*, resulting in neo-TADs that may bring skeletal enhancers in the non-deleted part of the *SULF1* TAD in proximity to genes in the adjacent TAD (*PRDM14*, *NCOA2*) and even into the next TAD (*EYA1*)(curved lines)

## Discussion

We describe a boy where cytogenomic and mate-pair mapping revealed two structural chromosomal alterations: aCGH identified a segmental loss at 15q12 of 21.9 kb considered to be pathogenic.

The segmental loss at 15q12 of 21.9 kb affects the *GABRA5* gene region, which has been associated with ASD [17]. *GABRA5* is a member of the Gamma-Aminobutyric Acid (GABA) receptor gene family, which are proteins involved in GABAergic neurotransmission in the mammalian central nervous system. GABA-A receptors are the site of actuation for several important pharmacological agents, including barbiturates, benzodiazepines, and ethanol [18].

Polymorphic Tandem Repeat (PTR) at the *GABRA5* locus have been described, including both deletions and duplications in the 15q11-q13 region, known to be methylated in Prader Willi and Angelman syndromes (MIM, 176,270; MIM, 105,830) [19, 20]. In turn, Butler et al. [21] identified a heterozygous de novo missense variant in the *GABRA5* gene (V294L; 137142.0001) in a 2-year-old boy with epileptic encephalopathy and developmental disorder.

As well, Hernandez et al. [22] identified heterozygous de novo mutations in the *GABRA5* gene (V294F, 137142.0002 and S413F, 137142.0003). The variation detected in our patient was considered pathogenic and compatibel with the ASD phenotype.

The gnomAD database contain variants originating from > 730,000 whole exome and > 75,000 whole genome sequenced unrelated individuals [23]. One specific feature of gnomAD is that the cumulative variant data reveal if a specific gene can tolerate loss-of-function variants. Thus, > 3,000 of the human genes are loss-of-function constrained (have a pLI-score > 0,9). *GABRA5* has an intermediate pLI of 0.79. *GABRA5* is specifically and higly expressed in the brain.

Mate-pair sequencing resolved the translocation breakpoints with a resolution of 303 bp within *PLPP3* on chromosome 1 and 193 bp within *SLCO5A1* on chromosome 8.

*PLPP3* encodes a member of the phosphatidic acid phosphatase (PAP) family. It is a membrane glycoprotein and a loss-of-function constrained gene with a pLI = 1. It is not related to a known clinical phenotype. It is not a brain-specific gene but is expressed in most tissues.

*SLCO5A1* (Solute Carrier Organic Anion Transporter Family Member 5A1) encodes a protein involved in cellular transport activity. Its expression is detected in numerous tissues, such as the brain, heart, skeletal muscle, ovary, and breasts, with highest expression in skeletal muscle and heart (MIM, 613,543). This gene has been suggested as one of the two candidate genes for Mesomelia-Synostoses Syndrome (MMS), alongside *SULF1*. However, it has a pLI = 0, indicating that heterozygocity for inactivating variants including the reported deletions and the present translocation breakpoint are likely not contributing to MSS. It is not or has as very low expression in the brain.

Likewise, *SULF1* with a pLI = 1 is not or has as very low expression in the brain.

Mesomelia-Synostoses Syndrome (MMS) is an autosomal dominant type of mesomelic dysplasia that comprises typical acral synostoses combined with ptosis, hypertelorism, palatal abnormality, congenital heart disease, and ureteral anomalies (MIM, 600,383). Only one report of a patient with MMS without a deletion in the 8q13 region has been described, with a monoallelic expression of *SULF1* in fibroblasts, but with no alterations in *SLCO5A1*, or in any other gene tested [11]. In contrast, five unrelated patients with MMS have microdeletions in the 8q13 region, varying from 582 kb to 738 kb in size but always involving *SULF1* and *SLCO5A1* [8–10, 24, 25]. Although both are expressed in skeletal tissues, *SULF1* is the candidate that might have a major influence with respect to triggering skeletal abnormalities. However, homozygous deletion of *Sulf1* results in milder skeletal defects in mice (reduced bone length, early ossification of vertebrae, and fusion of the vertebrae with tail vertebrae), but not the more severe spectrum including extra-skeletal features as seen in MSS [26]. Along with the general lack of loss-of-function point mutations in MSS, this suggest that *SULF1* haploinsufficency alone cannot fully explain the spectrum seen in MSS.

The human genome is organized into megabase-sized topological associated domains (TADs) [14] and chromatin loops [15, 16] that define cis-regulatory interactions. Disruption of this 3D-organization by structural rearrangements incl. deletions, duplications, inversions and translocations, may cause developmental disorders due to placement of ectopic enhancers in neo-TADs harbouring developmental genes [27; for a review, see ref. 28].

Importantly, the two genes deleted in MSS, *SULF1* and *SLCO5A1*, are situated next to each other but in separate TADs (Fig. 6). Hence, the MSS-associated deletions invariably remove the TAD boundary separating these two genes (Fig. 6). The TAD harbouring *SULF1* is enriched in evolutionary conserved non-genic elements which likely include skeletal and chondrogenic enhancers (cf. the *Sulf1-/-* mouse model) and part of this TAD (with their putative enhancers) are retained in all the MSS-deletions (Fig. 6). Chromatin loops are the underlying structures mediating these mostly intra-TAD regulatory interactions. Deletion of the *SULF1*/*SLCO5A1* TAD boundary opens for novel chromatin loops within the merged neo-TAD, which may extend to the candidate constrained genes *PRDM14* (pLI = 0.92) and *NCOA2 (NCOA2*: pLI = 1*)*, but also neo-loops that extend further into the next TAD harbouring the key developmental gene *EYA1* (pLI = 1). Hence, we propose ectopic enhancer adoption as an alternative and most likely explanation for MSS. Indeed, a position effect on neighbouring gene(s) was mentioned briefly as a speculative explanation in the original paper defining the 8q13 deletions in MSS [10].

## Conclusion

This article demonstrates once again the effectiveness of balanced structural rearrangements associated with clinical phenotypes as a tool for mapping and defining Mendelian disorders. Although the translocation is likely not contributing to the phenotype of our patient per se, the breakpoint together with the emerging data on the 3D- organization of the human genome has contributed to further delineation of a not fully understood monogenic disorder.

## Data Availability

All data generated or analyzed in this study are included in this published article.
